# The Endless Sources of Hepatocellular Carcinoma Heterogeneity

**DOI:** 10.3390/cancers13112621

**Published:** 2021-05-26

**Authors:** Marina Barcena-Varela, Amaia Lujambio

**Affiliations:** 1 Department of Oncological Sciences, Icahn School of Medicine at Mount Sinai, New York, NY 10029, USA; marina.barcena-varela@mssm.edu; 2 Liver Cancer Program, Division of Liver Diseases, Department of Medicine, Tisch Cancer Institute, Icahn School of Medicine at Mount Sinai, New York, NY 10029, USA; 3 The Precision Immunology Institute, Icahn School of Medicine at Mount Sinai, New York, NY 10029, USA; 4 Graduate School of Biomedical Sciences at Icahn School of Medicine at Mount Sinai, New York, NY 10029, USA

**Keywords:** hepatocellular carcinoma, heterogeneity, molecular mechanisms, risk factors, microenvironment

## Abstract

**Simple Summary:**

Tumor heterogeneity in liver cancer is a major contributor to the high lethality rate found in patients suffering from this disease. The therapeutic outcomes are drastically affected by this heterogeneity, which complicates patient stratification and response prediction. Better understanding of all the factors that can contribute to this heterogeneity will be critical to improve our understanding of liver cancer, in order to optimize the outcome of patients.

**Abstract:**

Hepatocellular carcinoma (HCC) represents a global health problem. The incidence keeps increasing and current therapeutic options confer limited benefits to the patients. Tumor heterogeneity plays a central role in this context, limiting the availability of predictive biomarkers and complicating the criteria used to choose the most suitable therapeutic option. HCC heterogeneity occurs at different levels: within the population (inter-patient heterogeneity) and within tumors from the same patient (intra-patient and intra-tumor heterogeneity). Experts in the field have made many efforts to classify the patients based on clinicopathological characteristics and molecular signatures; however, there is still much work ahead to be able to integrate the extra-tumor heterogeneity that emerges from the complexity of the tumor microenvironment, which plays a critical role in the pathogenesis of the disease and therapy responses. In this review, we summarize tumor intrinsic and extrinsic sources of heterogeneity of the most common etiologies of HCC and summarize the most recent discoveries regarding the evolutionary trajectory of liver cancer cells and the influence of tumor-extrinsic factors such as the microbiome and the host immune system. We further highlight the potential of novel high-throughput methodologies to contribute to a better understanding of this devastating disease and to the improvement of the clinical management of patients.

## 1. Introduction

Liver cancer represents a major health problem, causing more than 800,000 deaths annually worldwide [1]. The most frequent type of liver cancer, which represents the second leading cause of cancer-related mortality, is hepatocellular carcinoma (HCC) [2,3]. Although HCC treatment has greatly improved over the past decades, HCC patients are generally diagnosed at advanced stages and ineligible for curative ablative therapies. There are several non-surgical therapeutic options available to treat HCC such as chemoembolization, radiofrequency ablation, or radiation therapy. However, liver resection and liver transplantation remain the main curative therapy options [4,5,6]. Concerning systemic treatment options, there are several therapies approved for advanced HCC patients, including the multikinase inhibitors sorafenib, regorafenib, lenvatinib, carbozantinib; the anti-angiogenic antibody ramucirumab; the immune checkpoint inhibitors nivolumab and pembrolizumab; and the combination therapies atezolizumab (anti-PD-L1) + bevacizumab (anti-VEGFA), and nivolumab (anti-PD-1) + ipilimumab (anti-CTLA4) [7,8,9,10,11,12,13]. Although these agents improve the survival of patients, with objective response rates of around 30% and survival benefits of several months, there is still room for substantial clinical improvements. One plausible explanation for the unsatisfactory clinical results is the high inter-patient heterogeneity among HCC patients and the lack of validated biomarkers that can help select the patients that are most likely to respond to each therapy. 

In addition to the vast inter-patient heterogeneity, HCCs are characterized by high levels of intra-tumor heterogeneity, which entail that tumor cells within a tumor are phenotypically different [14,15]. Moreover, approximately 70% of HCC patients develop early-stage recurrence following curative resection [16,17] and many studies have revealed that an elevated proportion of these recurrent tumors harbor clones that are different from the primary tumor, indicating de novo development or synchronous tumors rather than residual tumor or intrahepatic micrometastasis, contributing to intra-patient tumor heterogeneity within a single patient [14,18,19]. Most importantly, the unique etiology of HCC, associated with liver damage in 90% of all the cases, gives rise to high extra-tumor heterogeneity associated with the specific liver damaging mechanism and the host microbiome and immune system. In this review we will summarize the heterogeneous molecular profiles of HCC tumors and the most relevant contributing factors to HCC heterogeneity. We will also provide an update on the understanding of this diversity and how pertinent this knowledge is for translational research, as the consequences of this heterogeneity have a direct impact on the response to therapies against HCC. 

## 2. Inter-Patient Heterogeneity in HCC

The high inter-patient heterogeneity in HCC limits the availability of predictive biomarkers, complicates the development and clinical implantation of targeted therapies, and contributes to the poor outcome of the patients. The dissection of the contribution of each tumor-extrinsic and tumor-intrinsic factor in the process of hepatocarcinogenesis may help in the future to discover novel biomarkers and better define prognosis in patients.

### 2.1. Diversity of Tumor-Extrinsic Factors: Extra-Tumor Heterogeneity

HCC is a complex malignancy that can be triggered by many different factors including hepatic viral infections, alcohol abuse, and diet-induced metabolic disorders. The different etiologies can promote various oncogenic pathways and most liver tumors (up to 90%) develop in the context of underlying chronic liver disease (CLD) that is characterized by the accumulation of extracellular matrix (fibrosis), scar formation (fibrosis and cirrhosis), and chronic inflammation, which together constitute the perfect pro-oncogenic niche for tumor initiation and progression [19,20,21]. 

More importantly, the surrounding microenvironment greatly affects the phenotypic output of liver tumors, even of those presenting activation of the same oncogenic pathways, contributing to HCC heterogeneity and highlighting the relevance of all the factors involved in the malignant transformation of hepatocytes [22]. Microenvironmental factors, such as the host gut microbiota, have also been shown to play an essential role in liver tumorigenesis, and the precise mechanisms of this contribution are starting to be elucidated [23,24].

Among the different tumor-extrinsic factors, demography is a very relevant epidemiologic aspect to consider. Viral infections caused by hepatitis B virus (HBV) and environmental exposure to aflatoxin B1, for example, are the most frequent risk factors in Asia and Africa, whereas hepatitis C virus (HCV) infection, chronic alcohol intake, and metabolic syndrome are frequently related to HCC in Western countries [17,25]. Among the different hepatitis viruses, only hepatitis B and C viruses (HBV, HCV) have been demonstrated to cause HCC [26], whereas hepatitis D viruses (HDV) can be found in HCC patients that are co-infected with HBV. However, the contribution of HDV to the carcinogenic process is still not clear [27,28]. HBV infection presents its highest prevalence in Asia followed by Africa, while the epidemiological distribution of HCV infection is more globally spread, with Japan, Italy, and the United States among the countries with the highest prevalence [29]. The precise oncogenic mechanisms driven by hepatitis viral infections are not completely understood. Myriad molecular and cellular events related to viral injection have been characterized including intracellular DNA rearrangement and chromatin instability, promotion of mutations or overexpression by DNA integration (insertional mutagenesis), stimulation of proliferation, and activation of inflammatory signals leading to chronic inflammation within the liver. This persistent hepatocyte inflammation is one of the main contributors to premalignant stages in the liver [30]. Additionally, viral infections by HBV and HCV have been associated with immune escape and exhaustion of virus-specific T-cells as a result of sustained antigenic stimulation [31,32], shaping a liver immune microenvironment that can greatly influence tumor development and progression.

The exposure to environmental carcinogens has also been linked to the emergence of specific genetic alterations. The best known example, aflatoxin B1 (AFB1), is a mycotoxin that has been found in contaminated food, mainly in Asia and Africa, and is associated with a specific genetic signature of HCC characterized by a high rate of C > A mutations and the R249S specific mutation in the tumor suppressor TP53 [33,34]. In addition, single nucleotide polymorphisms (SNPs) of glutathione s-transferase enzyme gene variations GTSM1 and GSTT1, genes involved in carcinogen detoxification, are associated with a high risk of HCC in patients exposed to different genotoxic contaminants [35,36].

More prevalent in Western and developed countries are HCC cases associated with alcohol abuse and metabolic syndrome, although these etiologies are found globally. The incidence of alcoholic liver disease (ALD) and, specially, non-alcoholic fatty liver disease (NAFLD), have greatly increased across most developed countries during the last decades [3]. NAFLD is the most prevalent liver disease worldwide [3,37] and presents about 60% of probability to evolve to non-alcoholic steatohepatitis (NASH), a more progressed stage of CLD with a high risk of developing into HCC [37,38]. There is an emerging health concern in this context, a consequence of the excessive caloric intake and sedentary lifestyle leading to the obesity epidemic and metabolic syndrome in our society. Characterized by the accumulation of fat or steatosis in the liver, hepatocyte damage, and inflammation inherent to NASH, the pathogenesis of this disease is not fully understood. Lipotoxicity is known to promote the metabolic reprogramming in hepatocytes, favoring the accumulation of potential toxic metabolites that, combined with the inflammatory microenvironment and liver regeneration, contribute to DNA instability in NAFLD and NASH. Significant DNA damage responses and oxidative stress have also been found in these patients, which can also contribute to paving the way for tumor initiation [39,40].

Excessive alcohol intake can lead to alcoholic liver disease (ALD), and this is another prevalent diet-derived risk factor for HCC. The carcinogenic process in these patients, similar to that in NAFLD patients, has been linked to increased production of reactive oxygen species (ROS), changes in metabolic pathways, increased inflammation, and cell death along with impaired immune response [41]. Those patients with alcoholic HCC present a worse prognosis than those with non-alcoholic HCC and this is in part due to the impaired surveillance and late diagnosis but also to the poor compliance linked to this group of patients.

Related to diet-derived risk factors, there is a growing number of studies suggesting a strong contribution from host microbiome dysbiosis. Dysbiosis refers to changes in microbiota and, therefore, changes in microbial metabolites, that commonly co-occur with defects in the gastrointestinal barrier that facilitate the translocation of microbial products into the portal circulation. Patients with NASH [42,43] or cirrhosis [44,45] present gut dysbiosis, and a strong association was found between the host microbiome profile and alcohol intake [46]. There is also increasing evidence demonstrating a critical role of the gut microbiome and the microbial metabolites in hepatic tumorigenesis [24,45], including the impairment of anti-tumor immunity [23,24]. However, little is known about the association between bacterial diversity and tumor-intrinsic factors. The possibility of the gut microbiome influencing tumor growth has attracted the attention of many experts who seek to develop microbiome-based biomarkers and microbe-based therapies.

Finally, another relevant tumor-extrinsic factor to be mentioned in this review is the immune system, which is patient-specific with a unique genome and expression patterns, plays a pivotal role in tumor growth, and therefore contributes to inter-patient heterogeneity. Special attention has been paid to the study of the tumor immune microenvironment in the last years due to the emergence of immune-based therapies as effective first-line systemic treatment options against HCC. Moreover, the availability of high-resolution comprehensive analyses has enabled the immune profiling of HCC tumors despite their diversity and complexity. Studies of the interactions between tumor cells, immune cells, and non-immune stromal cells have shown different patterns across the different tumor subtypes, demonstrating clinicopathological significance and inter-patient, but also intra-tumoral, heterogeneity [47,48,49,50,51]. The anti-tumor immunity refers to both innate and adaptive immune responses, which can lead to tumor control and involve many different immune cell subtypes and molecular pathways. By estimating immune cell infiltrates from gene-expression data, approximately one quarter of HCCs have been defined as “immune-class”, characterized by markers of anti-inflammatory responses. Those cancers presenting enhanced cytotoxic T-cell and interferon (IFN)-related genes are subdivided as “active-immune-class” HCC (about 10%), associated with better prognosis, whereas an “exhausted immune-subclass” (about 10%) is defined by moderate expression of cytotoxic T-cell signatures and significantly high expression of exhausted T-cell signatures [52,53,54]. Emerging studies focused on deciphering the contribution of single immune cells, inflammatory signatures, and functional states of the immune populations are revealing a more complex dynamic biodiversity in the hepatocellular microenvironment [48,49,50,51,53,55]. This information may in the future facilitate the stratification of patients to support relevant clinical decisions in which the status of the anti-tumor immunity plays a pivotal role. Concerning the remaining 75% of HCCs that are not included in the “immune class” and present an immune-desert phenotype, they also represent a highly heterogenous group where immune evasion is the leading actor. Tumor cells can impair anti-tumor immunity through various mechanisms that are still being uncovered [56,57]. As an example, our laboratory has contributed to this field by functionally validating the role of β-catenin activation promoting immune escape and resistance to immunotherapy in HCC. Using genetic customizable mouse models of HCC [58], Ruiz de Galarreta et al. demonstrated that activated β-catenin pathway promotes immune escape by defective recruitment of dendritic cells and consequent impaired T-cell activity [59]. This study suggested that specific genetic alterations found in HCC might be able to trigger different mechanisms to avoid anti-tumor immune responses. Identifying these associations would be very useful to better understand the molecular basis involved in the tumor phenotypes and predict at which level the host immunity impairment occurs, offering potential biomarkers or paving the way to design novel targeted therapies. Taken together, improved knowledge of the immune landscape of the patients and its associations with genetic alterations of the tumors might be able to overcome the limitations found in terms of effectiveness in HCC patients and potentially identify those cases more susceptible to responding to immune checkpoint inhibitors that are currently available in the clinic.

### 2.2. Tumor-Intrinsic Factors: The Genetic and Epigenetic Landscape of HCC

During hepatocarcinogenesis, as well as during the carcinogenic process of other solid tumors, numerous genetic alterations emerge and accumulate during the different stages of the disease, resulting in unique cancer genomes. An accurate landscape of the genetic alterations in HCC has been established thanks to the cumulative data from high-throughput sequencing efforts for large numbers of samples from patients. In order to improve the understanding of the molecular basis of HCC, several molecular subclasses presenting distinct oncogene signaling pathways and recurrent mutations have been established [22,60,61,62,63,64].

Unlike other tumor types, HCC rarely appears as a consequence of monogenic syndromes. Exceptionally, HCC has been seen in patients with APC germ-line mutations or presenting rare monogenic metabolic diseases such as hemochromatosis (HFE1 gene alterations), Wilson disease (ATP7B gene alterations), tyrosinemia type I (FAH gene alterations), cutanea tarda (UROD gene alterations), a1 antitrypsin disease (SERPINA1 gene alterations), von Gierke disease (G6PC gene alterations), or diabetes of the young type 3 (MODY3 or hepatocyte nuclear factor 1A; HNF1A gene alterations) [22,65]. In addition to these rare cases of monogenic-disease-induced HCC, hepatocellular adenoma (HCA) is a rare benign tumor developed from hepatocytes in the context of a non-damaged normal liver. These tumors, extensively studied by the laboratory of Prof. Zucman-Rossi, are associated with pre-menopausal young women, linked with specific genetic signatures, and divided into three molecular subclasses defined by (a) inactivation of the transcription factor HNF1A, (b) activation of the WNT/β-catenin pathways by CTNNB1 mutations, or (c) activation of the IL6/STAT3 pathway by somatic mutation of IL6ST, GNAS, or STAT3 [62].

Most of the polymorphisms associated with HCC development are related to specific etiologies in the context of CLD, highlighting the close relation between the different environmental factors and the genetic background in the liver [22]. Although it is hard to determine the dominant reprogramming events involved in hepatocarcinogenesis, there are some clear examples of these (micro) environmental–genetic correlations. HBV infection, for example, induces chromosome instability and insertional mutagenesis [64,66] and has been associated with TP53 mutations [60], whereas patatin-like phospholipase domain-containing protein 3 (PNPLA3) polymorphisms (coding a lipase involved in triacylglycerol hydrolysis) are strongly associated with the chronic liver disorders ALD [67] and NAFLD [68,69]. Transmembrane 6 superfamily member 2 (TM6SF2) variants are also strongly associated with the presence of NAFLD and have been correlated with the severity of steatosis, NASH, and cirrhosis [70,71]. Other examples are mutations in CTNNB1 gene encoding for β-catenin protein, which have been correlated with alcoholic HCC [60]. In addition to differences based on different genetic alterations, the sequential appearance of the genetic alterations can be different depending on the surrounding etiology of HCC and the tumor stage. In normal hepatocytes undergoing hyperproliferation (HCA), CTNNB1 mutations usually occur prior to the telomerase reverse transcriptase (TERT) promoter mutations, whereas in the case of HCC development in cirrhotic liver (accounting for most of the cases), TERT promoter mutations are observed first, in pre-cancerous stages [22,60,72]. However, alterations in some fibroblast growth factor family genes such as FGF3, FGF4, FGF19/CCND1 amplification, and TP53 and Cyclin Dependent Kinase Inhibitor 2A (CDKN2A) mutations and deletions, appeared at more advanced stages [60]. Despite the identified risk-factor-specific mutational profile, there is still tremendous heterogeneity within each etiological group and the high diversity of the altered pathways found in HCC still hinders the stratification of the patients based on their molecular signature.

The so-called “genomic landscape of HCC” has been rigorously characterized in the work published in 2015 by Schulze and colleagues [60], which identified the major pathways recurrently altered in HCC (in more than 5% of patients), involving mutations in telomerase (60%) [72], WNT/b-catenin (54%), PI3K/AKT/mTOR (51%), TP53/cell cycle (49%), MAP kinase (43%), hepatic differentiation genes (34%), epigenetic regulation (32%), chromatin remodeling (28%), oxidative stress (12%), I16/JAK/STAT (9%), and transforming growth factor-β (TGFβ) (5%) pathways [22,60]. TERT, the enzymatic subunit of the telomerase, is not expressed in mature healthy hepatocytes, leading to inactivated telomerase function in the adult liver. HCC presents reactivated telomerase activity mainly due to somatic TERT promoter mutations (54–60%) [72,73]. TERT mutations have been considered as the earliest recurrent somatic alterations during malignant transformation as mutations in the gene can be found in premalignant lesions and cirrhotic livers, and then the frequency of the mutations increases drastically in early HCC to remain stable in the progression of the disease until advanced stages. Thus, TERT promoter mutations have been proposed as a good candidate biomarker of high risk for full malignant transformation and progression to advanced HCC. However, additional hits in other cancer-related genes are necessary for tumor development [22]. Among the oncogenic pathways altered in HCC, the WNT/b-catenin is the most frequent pathway, overstimulated by activating mutations in CTNNB1 [74] or inactivating mutations/DNA methylation in AXIN1 and in adenomatous polyposis coli gene (APC), respectively [22]. The PI3K/AKT/mTOR pathway is also activated in some HCCs by mutations in different genes from the pathway or upstream effectors, and alterations in this pathway have been linked to resistance to sorafenib [75,76], the most used systemic drug against HCC. Other important carcinogenic pathways deregulated in HCC involve somatic mutations in the tumor suppressor gene TP53 [34,60]; alterations to TP53 have been found in HCC patients with viral infection (HBV) and also chemical exposure.

In addition to genetic alterations, aberrations in the epigenetic machinery are commonly observed in HCC [77,78]. Epigenetic modifications participate in the regulation of gene transcription and are critical for maintaining cellular identity. Alterations in epigenetic information and aberrant expression and activity of epigenetic enzymes participate in the process of malignant transformation from preneoplastic stages. The increased relevance of epigenetic alterations in HCC emergence is in part due to their potential role as prognostic and diagnostic biomarkers [77,79]. The first epigenetic abnormality described in HCC was the dysregulation of DNA methylation, including genome-wide hypomethylation and region-specific hypermethylation, which can already be found in pre-neoplastic stages during CLD processes [78,79,80,81,82,83]. Genome-wide DNA methylation patterns in human liver from patients with different CLD can distinguish different stages of NAFLD, ALD, and liver fibrosis [79,81,82,83] and have prognostic value for HCC development or recurrence. Furthermore, methylation-based signatures have been generated in HCC patients [77,84,85], associated with different outcomes and molecular subclasses, and thus demonstrate its prognostic capacity. However, the dysregulation of epigenetic modifiers and their role in hepatocarcinogenesis is being actively investigated. For example, mutations of chromatin remodeling modifiers from the SWI/SNF complexes and mixed lineage leukemia gene family (MLLs) have been identified, including mutations in ARID1A (7–17%) [86], ARID2 (3–18%) [87], MLL (3–4%), MLL2 (2–3%), MLL3 (3–6%), MLL4 (2–3%), and MLL4 (10%), which represent some of the most common epigenetic mutations found in HCC samples. As occurs with the alterations found in the DNA methylation patterns, the altered expression and activity of the epigenetic enzymes appear progressively during CLD and hepatocarcinogenesis, contributing to the reprogramming of the chromatin architecture and the transcriptional landscape [78,83].

Summarizing this section, it can be clearly concluded that, contrary to other solid tumors, HCC development is not determined by the dysregulation of one specific molecular pathway but of many. Prediction of patients’ outcome or prognosis and therapeutic decisions based on the molecular features of the disease cannot be limited to the unique presence of specific genetic or epigenetic alterations, but to the combination of all the altered pathways that usually emerge along the chronic process until tumor development and that can be subjected to changes during the tumor progression.

### 2.3. Tumor-Intrinsic Factors: Molecular Subclasses in HCC

As explained above, genetic alterations in HCC are found in a variety of cancer driver pathways with a highly heterogeneous pattern of alteration among patients [60,73,88]. The main challenge is to classify the different molecular profiles of HCC, in order to stratify the patients and more accurately define the treatment options and prognosis. However, subclassifications based only on genomic profiling do not encompass all the factors that contribute to the phenotypic heterogeneity observed in tumors, either because they do not integrate other factors, such as tumor-extrinsic factors, or due to the complexity and contribution of the possible combinations of mutations found in this cancer. There are 2 major subgroups of HCC tumors accurately defined by different biological processes: the proliferation and non-proliferation subclasses [89]. Although this classification is mainly based on the gene expression profile of the tumors, it is strongly associated with clinical and pathological features of the patients and their surrounding etiology, thus integrating different sources of information to define clinical outcomes [22]. On one hand, the proliferation subclass accounting for around 50% of patients is enriched in signals related to cell proliferation and cell cycle progression and is generally associated with a more aggressive phenotype and poor outcome. The activated signaling cascades found in this group and related to proliferation and cell survival include MET or hepatocyte growth factor receptor (HGFR) pathway [90], TGFβ signaling, insulin-like factor I (IGF) pathway [91], RAS/mitogen-activated protein kinase pathways [92], and AKT/MTOR signaling pathway [93], among others. Enrichment in the expression patterns associated with stem cell features (e.g., NOTCH) [94] and markers of progenitor cells (e.g., epithelial cell adhesion molecule) [95], also found in this subclass, indicate a moderate/poor cell differentiation state that correlates with more aggressive clinical behavior [96,97]. Another characteristic aspect of this subgroup is that tumors present higher rates of chromosomal instability due to aberrant epigenetic changes. In fact, there is a defined DNA methylation-based prognostic signature [77] that correlates with signatures of progenitor cells and poor survival. On the other hand, the non-proliferation subclass is characterized by a well-differentiated phenotype, with normal hepatocyte-like features. This subgroup of HCC is less aggressive and correlates with low levels of a-fetoprotein (AFP), the most widely used biomarker to detect HCC, contrary to the high levels found in the proliferation subclassified patients [22]. One subset of this group is characterized by the liver-specific activation of WNT signaling pathway (about 25%), mainly by mutations in CTNNB1 [98], while the other main subgroup is associated with immune response [99]. Overexpression of the epidermal growth factor receptor (EGFR) is also observed in the non-proliferation group and plays a critical role in the maintenance of the transformed phenotype of HCC cells [100]. Regarding etiologic factors, HBV-related HCC tumors are predominantly classified in the proliferation subclass, whereas HCV and alcohol-related HCC are more prevalent in the non-proliferation subclass.

Some groups of patients present HCC derived from the malignant transformation of hepatocellular adenoma, which is a rare benign hyperproliferation of hepatocytes [101,102]. HCA are subdivided into 4 groups based on their mutations in HNF1A (H-HCA), CTNNB1 (b-HCA), in the genes associated with activation of inflammatory pathways (IHCA), and an unclassified type of HCA [102,103]. The malignant transformation occurs in these cases differently depending on the genetic background. Particularly those HCA harboring β-catenin-activating mutations present the higher risk for malignant transformation, and TERT promoter mutations are involved in the last steps along with aberrant epigenetic alterations and chromosomal alterations [22,102].

In 2007, Boyault and colleagues [64] further subdivided HCC patients based on transcriptomic analysis and genotype–phenotype correlations. Their unsupervised clustering identified 6 robust subsets, G1–G6, that present high association with genetic alterations and clinical factors. Very briefly, G1 tumors are associated with a low copy number of HBV, aberrant overexpression of oncofetal genes, and AKT pathway activation. G2 tumors also present activated AKT pathways but are associated with a high copy number of HBV and mutations in PIK2CA and TP53. The third group, G3 tumors, also harbors mutations in TP53 along with the overexpression of genes controlling the cell cycle. G4, the most heterogeneous subgroup, includes TCF1-mutated HCC and HCA. G5 and G6 tumors are both related to activation of the WNT pathway (CTNNB1 mutations mainly), particularly G6 tumors, presenting higher activation of WNT than G5. The G6 group is also characterized by downregulation of E-cadherin expression and satellite lesions. In general, tumors from G1, G2, and G3 are associated with high rates of chromosomal instability, hyperproliferative phenotype, and worse prognosis in patients, whereas tumors from G4, G5, and G6 present chromosome stability and tend to present a better survival. In addition to this subclassification, two years later Hoshida and colleagues [63] published an integrative transcriptome analysis revealing three subclasses of HCC: S1, S2, and S3. These subclasses correlate with clinical parameters such as tumor size, cellular differentiation state, and serum AFP levels. The gene expression profile of the S1 group reflected an aberrant activation of the WNT pathway and overexpression of TGFβ target genes, whereas the S2 group was characterized by high proliferation and MYC and AKT activation. Finally, the S3 group signature correlated with hepatocyte differentiation and better survival.

Despite the advances in the stratification of HCC patients, above summarized and supported by the implementation of high-throughput analysis [104], the integration of data obtained from human studies and preclinical models remains necessary to accelerate the identification of robust predictive biomarkers and classification of candidate patients to increase the efficiency of targeted and immune-based therapies. In that regard, our laboratory has recently used the hydrodynamic delivery of genetic elements to study how different genetic alterations cooperate with each other to contribute to different expression patterns, inter-tumor heterogeneity, and distinct patient responses [58].

## 3. Intra-Tumor and Intra-Patient Heterogeneity

Intra-tumor heterogeneity is a feature frequently found in many solid tumors [105,106]. HCCs are frequently detected with a nodule-in-nodule appearance, where a subclone resides embedded in another [107,108]. Many other patients present multiple hepatic tumors that can develop from different clones (presenting more than one primary tumor simultaneously) or arise from a single original tumor via intrahepatic metastasis [109,110]. Tumor cells are subjected to a natural selection process within the tumors and the microenvironment that leads to clonal evolution and the coexistence of diverse clones in a single tumor. It is commonly thought that this intratumor heterogeneity is critically associated with the development of resistance against cancer therapies. In this sense, it can be hypothesized that a therapy might have an anti-tumor effect globally while inducing a positive selection in the most resistant tumoral subclones.

About 70% of the patients with HCC under curative treatment options develop recurrence, considered early-recurrence within two years of treatment or late-recurrence when it occurs after more than two years [16,17,111,112]. Although there are some studies indicating that genetic and DNA methylation patterns of HCC can be used to identify patients at high risk for recurrence [18], the origin and evolution of the relapsed tumors are still controversial. Traditionally, it was considered that early-relapsed tumors were derived from residual or intrahepatic micrometastasis rather than de novo tumorigenesis or synchronous tumors with variable clonality. However, in a study from 1989 [14] it was already shown that there is not a clear correlation between the time of appearance of the recurrence and the clonality of the recurrent tumors, an observation that was later supported by other researchers [18,113], that there is not a clear correlation between the time of appearance of the recurrence and the clonality of the recurrent tumors. More recently, an exhaustive clonality analysis at single-cell level of early relapsed HCC cohorts demonstrated that tumoral cells in relapsed tumors derived from a minor clone from the primary tumor and were characterized by a distinct tumor microenvironment that conferred enhanced immune evasion capabilities [113]. Focusing on evolutionary trajectories in liver cancer, Ding and colleagues [18] identified TP53, CTNNB1, and TERT mutations as founder drivers and convergent mutations in key drivers including TP53, TERT, CTNNB1, TSC2, JAK1, NOTCH1, FGFR3, ATRX, and RPS6KA3. These convergent mutations can favor each ancestor clone to experience parallel series of expansions, inferring an environmental selection that allows parallel or selective seeding of ancestor clones. These findings were in line with a previous work by Torrecilla S. et al. [114] where they described TERT, TP53, and CTNNB1 mutations as trunk alterations in HCC, meaning they are ubiquitously present across different regions of the same tumor and between primary and metastatic tumors in more than 85% of the cases studied. Ding et al. [18] further defined a co-evolutionary relationship between the genome and epigenome in HCC, highlighting a close interplay between genetic and epigenetic landscapes during malignant progression and pointing towards the potential of epigenetic signatures to define novel biomarkers to trace tumor evolution and progression. Recently, Losic and colleagues characterized the interactions of different components of the HCC ecosystem during cancer evolution by integrating RNA-seq, DNA-seq, TCR-seq, and SNP information from a dataset of multiregional samples from HCC patients. Their findings indicated that clonal evolution depends on different selection pressures that are primarily immune- and treatment-mediated [115], supporting the relevant role of the tumor microenvironment in clonal selection and intra-patient heterogeneity.

From a clinical perspective, it would be expected that high intra-tumoral heterogeneity and clonal diversity in HCC correlates with worse patient prognosis and limited therapeutic options, as this enhanced heterogeneity in the tumors would trigger worse therapeutic responses in addition to more susceptibility to developing targeted therapy resistance. In fact, it has been observed that those HCC patients with higher heterogeneity correlate with a worse prognosis and poor survival [115,116]; however, additional studies are necessary to decipher this association. Most importantly, both intra-tumor and intra-patient tumor heterogeneity contribute to vastly increasing inter-patient heterogeneity.

## 4. Clinical Implications of HCC Heterogeneity

**Figure 1:**
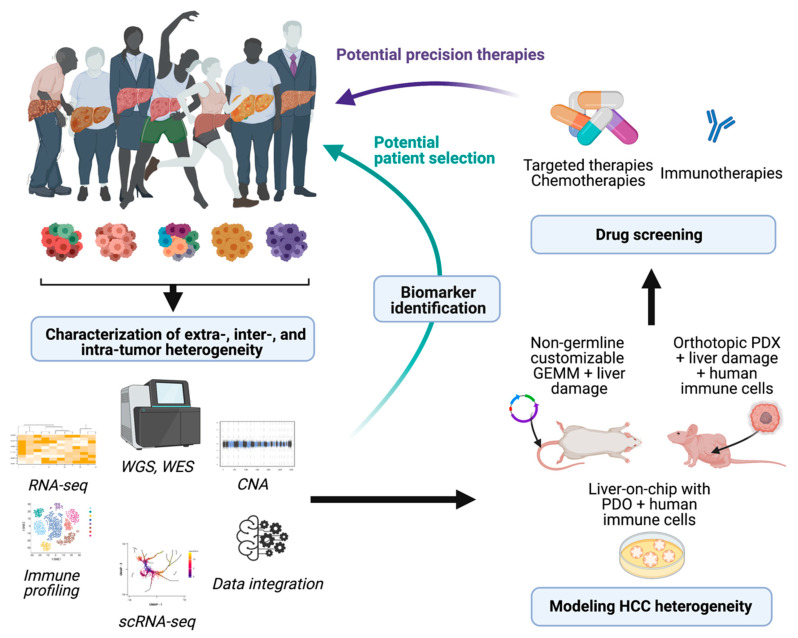
Potential implications of dissecting HCC heterogeneity. Implementation of high-throughput analysis to characterize HCC heterogeneity can provide useful biomarkers and pave the way for the development of novel models of HCC. The derived animal models and in vitro screenings that accurately recapitulate the complexity of this disease might represent powerful tools to test different drug sensitivities and potentially impact on the clinical management of patients. WGS, whole genome sequencing: WES, whole exome sequencing; CNA, copy number alteration; scRNA-seq, single-cell RNA-seq; PDX, patient-derived xenografts; PDO, patient-derived organoids. Figure prepared with Biorender.

References to the clinical implications of liver tumor heterogeneity have been mentioned along the different aspects addressed in this revision. The devastating concerns associated with the inefficient therapeutic responses against liver cancer reaffirm the imperative need to study in detail the mechanisms driving heterogeneity, to standardize a better subclassification of the patients for clinical management. Regarding decisions about the treatment of patients with HCC, physicians commonly rely on the stage of the disease rather than its molecular singularities. However, as it has been extensively explained here, the phenotypic differences at the molecular level between patients within the same stage are abysmal. Characterization of molecular subtypes and/or oncogenic signatures have generally improved the patient outcome for several types of cancer. However, the molecular classification of HCC has not been implemented for managing patients. Due to this complexity, there are not clear inter-patient biomarkers that can predict the response to a specific therapy (with the exception of ramucirumab and high AFP levels), whereas the high incidence of intra-tumor heterogeneity can induce a positive selection in the tumor subclones that is hard to foretell. In this sense, the widespread implementation of transcriptomic profiling of tumors at single-cell resolution by performing single-cell RNA and DNA sequencing along with single-cell proteomic studies might provide a comprehensive view of the tumor microenvironment and tumor cell trajectories to more accurately provide stratification strategies for HCC patients. The implementation of this novel methodology along with the use of experimental systems to properly model and understand HCC heterogeneity will enable better therapeutic approaches, based on the identification of molecular signatures that foster the choice of one therapy over another (see Figure 1). Moreover, identification of high intra-tumor heterogeneity in patients could be decisive for the election of combinatorial therapies over single-based treatment options in order to avoid the emergence of resistance. Regarding tumor recurrence of HCC, the effects of therapeutic options can be critical as patients might have very different prognoses and/or can benefit from different therapeutic approaches; unfortunately, primary tumors are characterized in most circumstances. In fact, it might be necessary to re-biopsy recurrences, regardless of relapsed time of the patients, to establish clonal origin and make decisions about new potential therapies that might be very different from the therapies proposed in the first instance.

## 5. Conclusions

Altogether, from a clinical point of view, the introduction of standardized methods to assess tumor heterogeneity in liver cancer would most likely enable better therapeutic outcomes and improve survival prognosis. Finally, it would be ideal to implement novel tools to monitor, in real-time, the molecular changes and cancer clonal evolution in patients with HCC. The technical advances in the next decade will be critical to reduce costs and show feasibility.

## Data Availability

The data presented in this study are available on request from the corresponding author.
